# Anticipatory declarations in obstetric care: a relational and spatial examination of patient empowerment, institutional impacts and temporal challenges

**DOI:** 10.1093/medlaw/fwae032

**Published:** 2024-08-23

**Authors:** Aimee V Hulme

**Affiliations:** School of Law and Social Justice, University of Liverpool, Liverpool, L69 7ZR, UK

**Keywords:** anticipatory and contingent declarations, court of protection, enforced caesareans, mental capacity, obstetric care, pregnancy

## Abstract

Seeking an anticipatory declaration from the Court of Protection (CoP) to manage a risk of future loss of capacity in pregnant people during labour and delivery appears to be occurring more frequently. This article examines a growing case sample of recent CoP judgments in which anticipatory declarations have been sought and adopts a combined relational and spatial approach to question whether these types of anticipatory declarations empower patient autonomous choice, and to illuminate the complex web of relational, spatial, and temporal factors that hold influence over the way in which mental capacity law operates. Viewing such processes from both a patient and institutional perspective offers useful insights into the law’s normative workings, boundaries, and constraints, and ultimately points to conclusions on the (in)effectiveness of anticipatory declarations as a legal mechanism for dealing with the risk of a patient losing capacity in the future. Moreover, however, taking a broader, spatial view signals the challenges posed by these cases to mental capacity legislation itself. The justifiability of the binary construct of capacity/incapacity has been challenged by some writers in this field, and this article offers further reflection on the integrity of this binary through its discussion of anticipatory orders for pregnant people.

## I. INTRODUCTION

An anticipatory declaration from the Court of Protection (CoP) may be sought when it becomes apparent that a capacitous individual could lose capacity at some point in the future in relation to a specific decision. For instance, a National Health Service (NHS) Trust could apply to the CoP for an anticipatory declaration stating that it would be lawful to carry out a certain procedure if the patient loses capacity to consent to it. Since 2019, at least six applications with three in 2023 alone have been made to the CoP by NHS Trusts requesting anticipatory relief in the form of a contingent declaration,^1^ stating it would be lawful for them to carry out a caesarean section if the pregnant person^2^ lost capacity just before or during labour. Not only have these applications been made *specifically* in relation to this select group of people, but this has shifted from including only pregnant people detained under the Mental Health Act 1983 (MHA) to the inclusion of those who have presented only a ‘small risk’ of losing capacity.^3^ This is concerning, as it sends a message to any pregnant person under NHS maternity pathways that their rights to choose a preferred method of delivery can be contested and potentially disregarded. Yet, choosing how to give birth has been described as a ‘central tenet of maternity care’.^4^ The NHS 3-year delivery plan for maternity and neonatal services released in March 2023 has its first objective set for ‘care that is personalised’ in order to ‘give people choice and control’ over their treatment.^5^ The reality, however, is that pregnant people often do not feel that they can exercise this choice.^6^

The purpose of this article is to bring to focus an alarming trend in NHS Trusts applying for anticipatory relief from the CoP when a capacitous pregnant person appears likely to lose capacity during labour or delivery, and it does so by gathering data from a case sample and associated literature. The article adds to the growing recognition of a shifting dynamic of medical law^7^ in that giving attention to spatial, temporal, and agential factors can highlight the restrictive nature of particular legal processes and provide a richer backdrop for understanding mental capacity law moving forward.^8^ This article follows approaches adopted by Clough^9^ and examines whether the autonomous choice of these patients has been respected, but beyond this, it explores the spatial and temporal factors attached to such declarations to ultimately unveil the constricting nature and boundaries of mental capacity law. These constraints are highlighted through the recognition that the court is consistently having to employ creative techniques to manage the sensitive temporal nature of these anticipatory cases, whilst preserving the integrity of the established legal framework. As a result, the core binary construct of mental capacity legislation is at risk of being undermined and is exposed for questioning.

First, the article explores the justification of the use of anticipatory declarations from the patient’s perspective. The historic lack of respect afforded to pregnant people’s autonomous choice in childbirth is briefly discussed paving the way for an analysis of the weight attached to both the status of the foetus and the weight given to the patient’s wishes and feelings in each case. Secondly, the article goes on to view these declarations through an institutional lens and considers the space in which NHS Trusts find themselves when the potential for ‘future incapacity’ and lack of future consent to a specific procedure becomes apparent. It is noted that the overhanging threat of liability claims against medical professionals influences the legal processes that emerge. However, this review shows that there is a varying judicial approach in the CoP as to the use and functionality of anticipatory declarations to manage anticipated future loss of capacity, resulting in a lack of certainty for NHS Trusts, which is clearly seeking guidance in this space. The court as an institution is also considered in terms of how hearings are held, the time they are held, or who attends the hearing to highlight how these factors can alter the course of events, particularly in that the patients in each case were not heard from directly in court. This then leads into a discussion on the temporal challenges that have come to the foreground when exploring these types of contingent declarations. The temporality in a situation whereby a pregnant person is in their latest stages of pregnancy and may possibly lose capacity just before or during delivery creates delicate obstacles to navigate. A review of the case sample reveals a choice for medical professionals in these circumstances either to wait and see if the situation becomes an emergency in the context of the Mental Capacity Act 2005 (MCA), thus triggering emergency provisions, or to seek an anticipatory declaration to intercept any future liability issues. The core issue, however, is that the applications for these types of decisions push the courts into counterbalancing these contextually sensitive temporal factors whilst remaining within the jurisdiction of mental capacity legislation. This proves a difficult task, but it is a significant one that this article aims to bring into focus in order to highlight the complexities of marrying these anticipatory cases with the MCA framework.

## II. THE CASE SAMPLE GATHERED

Two separate searches for the terms ‘anticipatory declaration’ and ‘anticipatory order’ were undertaken on Bailii and Westlaw to gather reported judgments that heard applications for anticipatory declarations in relation to future anticipated loss of capacity. Out of the initial search results, nine cases were found to be relevant,^10^ with the earliest reported in 2019. The discussion will focus, however, on six of the nine cases, as these involve applications for anticipatory declarations in relation to obstetric decisions because of a risk of the capacitous pregnant women losing capacity just before or during labour. These six cases are *United Lincolnshire Hospitals NHS Trust v CD*,^11^  *Guys And St Thomas NHS Foundation Trust (GSTT) & Anor v R*,^12^  *North Middlesex University Hospital NHS Trust v SR*,^13^  *North Middlesex University Hospital NHS Trust & Anor v MB*,^14^  *The Shrewsbury and Telford Hospital NHS Trust v T & Anor*,^15^  *Somerset NHS Foundation Trust v Amira*.^16^ The remaining three reported cases involve applicants seeking anticipatory declarations relating to care and treatment arrangements,^17^ care and contact with others,^18^ and care and diabetes treatment.^19^

Although admittedly a small sample, it is notable that two-thirds of the reported anticipatory declaration applications relate to pregnant women assumed to have capacity.^20^ The apparent issue in all of these six cases was a concern that the patients would lose capacity to consent to a caesarean section closer to the time of delivery. This raises significant questions, the main one being: Why have anticipatory declarations primarily been sought for this group of patients in these circumstances? Hundreds of life-saving operations are performed each day on many different people, some presumably with capacity issues and who may possibly lose capacity before the operation. Yet, it is those potentially withholding consent for a caesarean section that have come before the CoP under the justification of a future lack of capacity. Interestingly, however, out of the six cases, the judges in the first three granted the anticipatory declarations sought,^21^ but the judges in the last three did not.^22^ Out of the three applications that were not granted, two were denied because the judges held that the patients already lacked capacity.^23^ In the remaining case, the judge found the risk of future incapacity to be too small, so an anticipatory declaration was not deemed appropriate.^24^ The case sample, therefore, highlights that there is not necessarily an increase in the number of anticipatory declarations being issued, but there is an increase in the number of anticipatory declaration *applications*. This further points to the need for an exploration of the issues not only from a patient perspective but also from an institutional one, as it is obvious that the space in which the NHS Trusts find themselves is prompting these applications to the CoP.

## III. ANTICIPATORY DECLARATIONS TO MANAGE FUTURE LOSS OF CAPACITY IN PREGNANT PEOPLE

### A. The Law

In 1992, Lord Donaldson confirmed that a person can withhold their consent to any medical treatment, even if it results in the death of that person,^25^ but added a caveat to this where ‘the choice may lead to the death of a viable foetus’.^26^ This exception has since been debunked, and it is thus now generally accepted in law that a pregnant person can withhold consent to medical treatment even if this results in the death of the foetus, meaning that any treatment enforced upon the person would constitute a battery.^27^ Yet, if a pregnant person is found to lack the mental capacity to make a decision regarding their medical treatment, such medical treatment could be enforced if it is in the best interests of that person.^28^ The MCA states that a person will lack capacity ‘if at the material time he is unable to make a decision for himself in relation to the matter because of an impairment of, or disturbance in the functioning of, the mind of brain’.^29^ To be unable to make a decision, the person must be unable to understand, retain, and use or weigh the information relevant to the decision, or communicate his/her decision.^30^ Fundamental principles set out at the beginning of the Act include that a person is presumed to have capacity unless this can be rebutted,^31^ that all practicable steps must be taken to help a person make their own decision before they are to be treated as unable to,^32^ and that an unwise decision is not to be treated as an incapacitous decision.^33^ Furthermore, case law has established that having a mental impairment is not synonymous with lacking capacity,^34^ despite the ‘because of an impairment’^35^ element in the MCA’s test for incapacity. Whilst the structure of the MCA is straightforward, the CoP has heard a multitude of challenging cases that have not fitted so clearly into the binary cookie-cutter mould of capacity/incapacity that the MCA assumes.^36^

One area in which the judiciary has felt the need to set out further guidance is in relation to obstetric decisions. Keehan J in *NHS Trust & Others v FG*^37^ listed four circumstances in which NHS Trusts would be required to make an application to the CoP regarding a pregnant person who may lack capacity. These are:

1. when the intervention proposed by the NHS Trust amounts to serious medical treatment;
2. when there is a real risk that the patient will be subject to more than transient forcible restraint;
3. when there is a serious dispute as to what obstetric care is in the patient’s best interests; or
4. when there is a real risk that the patient will suffer a deprivation of liberty.^38^

This guidance came after it was argued by the Official Solicitor in the case that there had not been ‘a full appreciation or understanding of… the planning… the procedures… the timing of an application… [and] the evidence required to support an application’ in recent preceding cases relating to a lack of capacity over obstetric decisions.^39^ However, as per the focus of this article, there appears to be a new legal trend in which NHS Trusts are making applications to the CoP, not only in the circumstances outlined above but also in relation to pregnant people who *have* the capacity to decide what happens to them during labour and delivery.

### B. Anticipatory declarations and pregnancy

*In making a declaration that is contingent upon a person losing capacity in the future, the Court is doing no more than emphasising that the anticipated relief will be lawful when and only when P becomes incapacitous*.^40^

In its simplest form, an anticipatory declaration could appear to be an uncomplicated solution to a difficult problem. *United Lincolnshire Hospitals v CD* and *GSTT v R* were the first two reported cases that heard applications for anticipatory declarations. In both cases, the CoP stated that it was lawful for the respective NHS Trusts to carry out caesarean sections on the capacitous pregnant patients involved if they lost capacity before or during labour.^41^ Hayden J in *GSTT v R* clarified that the power to make such anticipatory declarations lay in Section 15(1)(c) of the MCA in that the CoP has the power to make declarations as to ‘the lawfulness or otherwise of any act done, or yet to be done, in relation to that person’.^42^ The judge stated that the phrase ‘yet to be done’ ‘contemplate[s] a factual scenario occurring at some future point’ and that ‘it is apt to apply to circumstances which are foreseeable as well as… those which are current’.^43^

Commentary on this justification of anticipatory declarations is scarce, but the main criticisms are summarized eloquently by Fovargue, observing that this particular reading of Section 15 undeniably conflicts with the Act’s other key sections along with its core principles.^44^ Specifically, Hayden J’s reliance on the phrase ‘yet to be done’ contradicts Section 2(1) of the MCA, which makes clear that capacity is both time and issue specific in that the question of capacity is being asked ‘in relation to a matter… at the material time’.^45^ In effect, an anticipatory declaration requires confirmation of capacity on a matter at an ‘*unknown* time in the future… for *unknown* reasons’ and, consequently, a best interests determination ‘at that *unknown* point in time’.^46^ It is contradictory to explicitly determine incapacity before the ‘material time’ has even occurred. A decision on capacity would be relevant to, and dependent on, the events occurring at the material time, circumstances which cannot be predicted with absolute certainty. Rogers J’s comments in a recent case are to the same effect: ‘the court has no jurisdiction to regulate the activities of capacitous individuals whatever objectively might be regarded as being in their best interests or designed to promote their welfare’.^47^ Through a spatial lens, Clough has argued that by making determinations of future capacity, ‘the spectre of the legislation is brought forward’,^48^ and the power dynamics afforded to medical professionals are brought ‘to a prior moment’ or ‘into the pre-labour space’.^49^

As a note of caution, however, Francis J in *United Lincolnshire Hospitals v CD* warned that anticipatory declarations should be issued by the CoP only ‘in exceptional circumstances’.^50^ However, there are doubts as to whether the circumstances in *CD* and in the subsequent cases are ‘exceptional’. For instance, presenting labour as the catalyst for a potential loss of capacity (as Francis J did in *CD*: ‘during labour her delusional beliefs may affect her judgment and she may again lose capacity to make decisions about the delivery for herself’)^51^ extends to a large group of people and perpetuates assumptions that ‘being in labour necessarily affects a woman’s capacity’.^52^ Underpinning this suspicion that a labouring person is liable to lose capacity are gendered, patriarchal undertones, which Villarmea describes as the ‘uterine influence’.^53^ Too often, those in labour are viewed through the preconceived, patriarchal lens that ‘obviously’ they will be in an ‘altered state and body of consciousness’ during labour, and thus, will not retain ‘full capacity’.^54^ This is of concern when a trend in applying for these anticipatory declarations appears to be in motion for pregnant people. Out of the six cases of interest, the first occurred in 2019, the next was heard in 2020, another was heard in 2021, and finally, the last three were heard in 2023. Statistically, the increase is noticeable, and contextually, it appears that the net to ‘capture’ those in anticipatory declarations has been cast even wider. The first two cases were related to pregnant women detained under the MHA,^55^ whereas in the subsequent four cases from the sample, none of the pregnant women were detained under the MHA.^56^ Fovargue warned of this in 2021, before these four further cases were decided, and stated that further seeking of anticipatory declarations insinuates that ‘childbirth choices of all pregnant women (regardless of their capacity) may again be at risk of challenge’.^57^

As Fovargue alludes to, there is an abundance of literature documenting poor quality of care for pregnant people, with one study on patient satisfaction whilst under maternity care reporting feelings of ‘no control’, ‘few choices’, and ‘little opportunity to influence decision-making’.^58^ This is unsurprising, considering that choice in childbirth has been described at separate points over a span of two decades as a ‘myth’,^59^ a ‘double-edged sword’,^60^ and an ‘illusion’.^61^ Baker and others argue that this illusion of choice comes from the expectation placed upon pregnant people to ‘behave and react in certain ways’ to ‘help keep childbirth within “normal” boundaries’.^62^ For instance, they argue that if intervention becomes an appropriate route in the eyes of healthcare professionals, pregnant people may feel pressurized to consent to that intervention.^63^ The ‘doctor-knows-best’ attitudes and the deference to expert medical knowledge, both in and outside of the courtroom,^64^ would certainly reinforce suggestions that there is a level of pressure on pregnant people to conform to what those overseeing their care feel is best. More alarming is that the literature referenced here comes from studies on those who, in theory, should be able to make their own decisions because they are regarded as having the capacity to decide. Halliday contends that this illusion of choice in childbirth is even more fictional for those with serious mental illnesses, as this ‘automatically marks [those individuals] out as different and requiring special treatment’.^65^ What this often means is that these individuals have their capacity called into question, and if found to lack capacity, decisions will be made for them in their best interests.^66^ The illusion of having options during childbirth is thus particularly heightened among those whose capacity is perceived as uncertain.

Nonetheless, the law appears clear: if a pregnant person has capacity, then they can refuse treatment, even if this results in their death or that of the foetus.^67^ If they lack capacity, a decision can be made for them in their best interests.^68^ However, the assessment of capacity is notoriously value-laden, and Fox and Lindsey have suggested that ‘women’s decisions are more likely to be overridden on the basis of incompetence [than men]’.^69^ In relation to the sample, out of the nine cases, eight of the patients identified as women, and six of the cases concerned labour and delivery decisions. Thus, in this context, Fox and Lindsey’s claim may have some traction. Feminist legal scholars have consistently argued that pregnancy does not diminish a person’s autonomous choice, agency, or rights.^70^ It is contended that it is ‘a perpetuated myth that pregnancy is “different” [to any other health issue]’,^71^ and to deny a pregnant person their autonomous rights would show a ‘sweeping disregard’ for the rights of a great number of people.^72^ The fact that these anticipatory declaration applications are being made in relation to pregnant people, however, suggests that pregnant people’s medical decisions are still singled out.

### C. From a patient perspective: curtailing choice in childbirth

All individuals in the case sample are adults with capacity who happen to be carrying a foetus. It would therefore be beneficial, and in line with a relational as well as spatial approach, to consider the patient perspective and, first, assess the case sample to determine to what extent the foetus’ presence explicitly or implicitly influenced the course of the cases, and secondly, whether the patient’s wishes and feelings were given any weight in the decisions. First, in *United Lincolnshire Hospitals v CD*, Francis J stated that the court should act in a way that promotes the welfare of the pregnant patient and ‘as part of that process… her unborn child’.^73^ The judge argued that ‘it is obvious… and as a matter of common sense that the loss of the baby would have a profound negative impact on [the patient]’,^74^ and as such stated that if a caesarean section was not performed, the foetus would be at risk of death due to excess amniotic fluid and the following consequences of that.^75^ The harm to the foetus therefore did likely influence the decision that the judge should make an anticipatory declaration. Similarly, in *GSTT v R*, Hayden J stated the excess amniotic fluid, also present in this case, risked the death of the foetus, and thus an anticipatory order was made.^76^ On best interests, Hayden J specifically stated that he believed saving an ‘unborn child’ was in the best interests of the ‘mother’, despite the fact that this went against the refusal to consent to a caesarean section.^77^ Again, it would be fair to state that the risk of harm to the foetus had bearing on the outcome of the decision to make an anticipatory declaration. In *North Middlesex University Hospital v MB*, although the patient was found to lack capacity anyway so an anticipatory declaration was not granted, Roberts J did discuss the health of the foetus and stated ‘I regard the safe delivery of [the patient’s] child as an intrinsic factor in a consideration of her own best interests’.^78^ However, in the final two cases, *Shrewsbury and Telford Hospital v T* and *Somerset v Amira* where anticipatory declarations were not granted, the respective judges did not discuss risks to the foetus. It may therefore be that when the mental capacity of a pregnant person is questioned and when intervention appears likely to be ‘necessary’, judges feel compelled to bring in foetal welfare to the conversation as a justification for said intervention. Furthermore, the use of the terms ‘unborn child’ and ‘mother’ to refer to the respective foetus and pregnant person, as Hayden J did in *GSTT v R*, further bolsters perceptions that a foetus requires protection from a pregnant person’s decisions and makes ‘judgments centring foetal welfare much more likely’.^79^ The evidence thus points to the conclusion that there are occasions when the foetus is treated as a ‘second patient’,^80^ sometimes inevitably curtailing the pregnant person’s choice of a particular mode of delivery.

Before reaching any final condemnatory conclusions about the significance for patients’ autonomy, it should be noted that their wishes and feelings found some expression in the final decisions. In *United Lincolnshire Hospitals v CD*, it was said that CD had ‘consistently expressed the wish to have a vaginal delivery’ but if this could not be achieved, then she would want a caesarean section under general anaesthetic.^81^ The final order that granted an anticipatory declaration stated that if CD were to lose capacity, it would be in her best interests to ‘try for a normal vaginal delivery’ but restraint could be used in order to administer general anaesthetic and perform a caesarean section.^82^ Consideration of CD’s wishes and feelings was therefore given in the final declaration. Likewise, in *North Middlesex University Hospital v SR*, SR was said to be ‘very clear that she wanted a caesarean’,^83^ which was held to be in her best interests in the anticipatory declaration.^84^ Furthermore, in *North Middlesex University Hospital v MB*, MB had expressed in her birth plan that she wanted a vaginal waterbirth, but in the event that she needed one, she would elect to have a caesarean section under spinal anaesthetic.^85^ The final best interests determination aligned with this birth plan.^86^ And lastly, in *Somerset v Amira*, Amira had a care plan (which she was involved in preparing when capacitous) that set out her delivery options in order of preference.^87^ Mostyn J at the end of his judgment stated: ‘I am fully satisfied that for Amira to be treated during the birthing process in accordance with the terms of the care plan is in her best interests and I so declare and order’.^88^

Whilst these cases are good examples of the courts reflecting patient’s wishes and feelings in the wording of the declarations, the same cannot be said for the case of *GSTT v R*, where R’s wishes and feelings were never sought in preparation for the hearing.^89^ In fact, it was only through a comment R made to medical staff, that a caesarean section would be ‘the last thing she would want’,^90^ that the judge was able to get an idea of her preferred mode of delivery. However, Hayden J appeared to justify his final decision that a caesarean section would be in R’s best interests^91^ by stating that ‘people use this phrase [“the last thing she would want”] loosely’ and that ‘P’s expressed wishes… are not regarded within the statutory framework as synonymous with P’s best interests’.^92^ Furthermore, the judge stated: ‘it seems reasonable to conclude that R would wish for a safe birth and a healthy baby’.^93^ This statement is particularly noteworthy as it highlights the central issues with making best interests decisions for pregnant people. First, is ‘a safe birth’ concerning safety for the foetus, the pregnant person, or both? Who determines how safety is framed in this context, and does it consider the psychological safety of the pregnant person? Secondly, by asking what would be ‘reasonable’ to conclude, the judge is arguably perpetuating gendered expectations and narratives of ‘good motherhood’ which often determines that ‘pregnant people’s behaviour need[s] to be self-sacrificial and altruistic’.^94^ By taking a more holistic, spatial, and institutional consideration of the judicial approaches to wishes and feelings, it becomes clear that the wishes and feelings of a pregnant patient are immediately viewed through a particularly narrow lens before they have necessarily been determined.

Notwithstanding these particular issues, the judgment in *GSTT v R* is an important example of the issues that can arise when not ascertaining a patient’s wishes and feelings directly, but hearing briefly of them reconstructed through the voices of others. The need to identify a patient’s wishes and feelings in good time was further magnified in *Shrewsbury and Telford Hospital v T*, where Lieven J focused more on the need for healthcare professionals to ensure that patients are given the option of making an advance statement of wishes and feelings,^95^ instead of ‘taking the draconian and properly exceptional step’^96^ of seeking anticipatory declarations. The judge expressed quite forcefully:

*T had indicated quite clearly, at a point when no one disputed her capacity, that she would enter into an advanced declaration about medical treatment. In my view that was a far more appropriate way to deal with a potential loss of capacity, rather than engaging the Court in making an invasive and draconian order. Such an approach protects the woman’s autonomy, in a way that an anticipatory declaration does not do*.^97^

Observing this through the perspective of the patient, it is refreshing to see a judge expressing the importance of the patient’s voice and autonomy in determining the trajectory of their own treatment. Hayden J in a recent case on the best interests of a pregnant person (but not related to anticipatory declarations) took an even more explicit autonomy-based approach and made a declaration that, although the patient lacked capacity, those involved in her care should help and support her to make her own decision on whether or not to have a termination, but she could not be forced to have one.^98^ Commentators have argued that this case represents an approach that truly supports the patient in exercising their legal capacity.^99^ Turning back to the case sample, whilst in four out of the six anticipatory declaration cases, the wishes and feelings of the person at the heart of proceedings *were* incorporated into the best interests decisions, this was done so through, as Lieven J put it, ‘invasive and draconian’ orders.^100^ It could not be said that the patients were supported in exercising their legal capacity here. This reflects the very core complexities that are entangled within these types of decisions, including the institutional role of healthcare professionals and the CoP. It is to these issues that this article will now turn.

## IV. THE INSTITUTIONAL SPACE: MEDICAL AND JUDICIAL PERSPECTIVES

Relational approaches to medical law^101^ have attempted to challenge the simplistic view of individual mental capacity by arguing that a person’s capacity does not come from the rational, cognitive thoughts or unencumbered functioning of oneself, but is affected by a multitude of contextual, social, and environmental influences.^102^ More specifically, the early liberal ideology of the legal subject asserts that having autonomy, and thus potentially mental capacity,^103^ equates to having self-governing, individual, and rational thoughts and desires.^104^ However, this has been contested by the recognition of how persons are ‘socially embedded… formed within the context of social relationships and shaped by a complex of intersecting social determinants, such as race, class, gender and ethnicity’.^105^ Clough’s position on spatial dynamics as an adjunct, rather than as an alternative, to relational theory goes further and recognizes that often relational theory tends to reinforce the existing boundaries of the law because such an approach looks at how relationships can affect a person’s mental capacity. Within this, the binary idea that a person either has or lacks capacity still exists. A focus on spatial, temporal, and agential factors, however, can challenge these existing structures.^106^ Within this approach, spatial dynamics is used to consider the role of institutions and the way in which they can influence legal processes. This can break down the power dynamics behind legal decision-making, which thus questions the power structures that compound the law and that are often seen as naturally existing as opposed to constructed for a particular purpose. A number of these cases have emerged since the publication of Clough’s work on spatial dynamics, and as such, this section will focus on anticipatory declarations from an institutional perspective by viewing the spaces in which these applications are brought to court.

A persistent literal and thus perceived threat of liability is acute in obstetrics and gynaecology,^107^ but with research finding no evidence of negligence claims in maternity care encouraging healthcare professionals to practice more safely.^108^ Instead, there are multiple reports of the threat of litigation causing risk-averse medical practice^109^ or an increase in overly paternalistic approaches.^110^ Given the fear of litigation looming over those working in obstetrics, it is understandable that these clinicians may grasp onto any potential solution when confronted with the prospect of a patient losing the capacity to consent to a caesarean section in the near future. Thus, if we view these cases through a broader, institutional lens, there is the sense that these applications for anticipatory declarations have become Trusts’ last-ditch attempt to provide them with some sort of liability protection because, as will be discussed in Section V, the most appropriate way forward for these types of cases is unclear. On a general note, case law clearly has a direct effect on how NHS Trusts approach future situations, for example, when to make an application to court, or what applications are made and so forth. Certainly, it would be fair to state that Francis J’s judgment from *United Lincolnshire Hospitals v CD*, (where an anticipatory declaration was made) signals to NHS Trusts in a similar position that this is a viable legal route to a solution. Therefore, the attitudes of judges sitting in the institutional position can have a trickle-down effect on the attitudes of healthcare workers on the ground, in another institutional position, and, as a result, have repercussions for patients under their care. It is therefore important to consider judicial perceptions of and interpretations of the use of anticipatory declarations to ‘manage’ anticipated future loss of capacity in order to fully grasp how these institutional perspectives interact and determine one another.

Despite the precedent set in the use of these contingent declarations, the case sample shows that consensus among judges on this legal avenue is far from complete. Francis J in *United Lincolnshire Hospitals v CD* and Gollop KC in *North Middlesex University Hospital v SR* issued anticipatory declarations with no further comments as to their ethical validity, implying that the judges had no issue with such orders.^111^ Whereas, Hayden J in *GSTT v R* agreed that anticipatory declarations were lawful, whilst describing them as ‘draconian’^112^ and ‘orders of a profoundly intrusive nature’.^113^ Likewise, Lieven J in *Shrewsbury and Telford Hospital v T* agreed that such declarations were legally permissible, whilst also observing that they were ‘draconian’ and did not ‘protect the woman’s autonomy’ in ways that advance decisions on medical treatment do.^114^ However, Mostyn J in *Somerset v Amira* expressed that he was completely against the use of anticipatory declarations, believing it was not in the Court’s power to issue them. Instead, in his words, Trusts should be relying on emergency provisions in Sections 5 and 6 to address situations where a person loses capacity during labour.^115^ Moreover, the Judge stated ‘the device of a proleptic declaration under s.15(1)(c) is in my judgment directly contrary not only to the wording of the Act, but also to its essential scheme’.^116^

The completely divergent views of judges create a lack of certainty for Trusts seeking guidance on the appropriate way forward. Here we can see how institutional power dynamics can shape and alter the situations in question. Of course, many judicial decisions made under the MCA are subject to value judgments because of the conflicting principles at stake in upholding a person’s wishes and feelings and providing them with a level of protection from harm.^117^ However, when a potential ‘solution’ emerges and judges have such contrasting opinions on it, this inevitably puts NHS Trusts in a difficult situation. Furthermore, even parties to proceedings are not wholly convinced of the virtue of such declarations. Counsel for both parties in a recent unreported case urged the judge to steer clear from issuing any anticipatory declarations as to the patient’s potential loss of capacity during labour.^118^ Counsel for the patient was noted to have asked the court not to use any anticipatory declaration ‘as a backdoor way to say she lacks capacity’, and likewise, counsel for the NHS Trust had stated: ‘I would forcefully caution against pursuing that path’.^119^ Notwithstanding, after Mostyn J’s comments condemning the legality of anticipatory declarations in *Somerset v Amira*, the Director of Clinical Law at the University Hospital Southampton Trust released a note stating: ‘there are clinical reasons to hope that anticipatory declarations will endure. Seeking legal authority in the midst of a clinical crisis provides anxious uncertainty’.^120^ It thus appears that, despite the judgment in *Somerset v Amira*, Trusts may be still in favour of applying for anticipatory declarations if they are in this state of ‘clinical crisis’. More importantly, however, it is noted that by reviewing these cases with the medical institution in mind, that is from the perspective of a healthcare provider, the power nuances, rationale, and logic behind seeking these anticipatory declarations are brought to the fore, and therefore can be challenged.

This section has so far considered the institutional perspective with regard to the healthcare provider, but of course, there is also the state-controlled institution of the court. The CoP was established to make determinations on individuals’ capacity and best interests under the MCA 2005 relating to both financial and welfare matters.^121^ It has previously been depicted as a ‘secret’ court, however,^122^ and its transparency has been subject to research and debate.^123^ Within this, the lack of participation of P^124^ in the CoP has also received a fair amount of commentary.^125^ The majority of the literature on this topic appears to focus more on the statistical data regarding P’s engagement with the court process,^126^ whereas recent work by Lindsey considers how the courtroom as a space can influence how justice is administered, because, as she states, court spaces ‘are not simply neutral places… they encompass buildings, technology, personnel, practices and procedures, which all have a historical and social context’.^127^ Lindsey evaluates how the physical design and procedural aspects of the court can alter the active participation of those involved in proceedings.^128^ As she argues, the fact that in some CoP cases, one party is an ‘arm of the state’, that being the NHS Trust, there is a ‘perception of bias… which can be hugely intimidating for the person at the heart of the case’.^129^

It is easy to find support for Lindsey’s claim. Not one P in the case sample was spoken to by the judge, nor did any participate in the hearings. Interestingly, in *North Middlesex University Hospital v SR*, it was reported that when handed the court documents prior to the hearing, P was ‘very clear that she did not want them and did not want to attend’.^130^ It would be reasonable to expect a person to feel overwhelmed in her position; SR was 36 weeks pregnant with her first child, with, in the judge’s words, a ‘turbulent and damaging’ past,^131^ and was being handed court documents full of legal jargon and about to face a hearing to determine whether she could choose the way she gave birth. Other commentators have discussed how the general hierarchy within the courtroom can negatively influence those who are the subject of proceedings, and how this is evident in the traditionally elevated physical positioning of professional bodies, such as judges and counsel, where they sit during proceedings, compared to those who are the subject of proceedings or non-judicial.^132^ Of course, these different individuals are restricted in terms of where they are allowed to sit; however, these negative impacts of the way the courtroom is structured are reflected in the fact that ‘P’ need not even be physically present in a CoP hearing. In such instances, this person becomes distant and envisioned in court through the accounts or evidence provided by, for example, the Official Solicitor or healthcare professionals who have met with P on a prior occasion. Consequently, those who have not met with P are given a version of that individual, entangled with subjective interpretations of them.^133^ However, in *GSTT v R*, R had not even met with her court representative prior to the hearing: ‘it was not possible for the Official Solicitor to speak with R to achieve greater clarity as to her wishes and feelings’.^134^ In this instance, the interpretations of P were gathered through medical evidence, namely two ‘short capacity assessments’ in the medical records, a statement by a Consultant Psychiatrist and a statement by a Consultant Obstetrician.^135^ It is difficult to accept that procedural justice could be achieved without hearing the voice of ‘P’. Many disability theorists engaging with mental capacity law have advocated for improved processes such as ‘the introduction of supports to enable people to participate effectively in proceedings designed to administer justice’.^136^ However, looking from the institutional perspective of the court, and in relation to the case sample, in five out of the six cases, the hearings were urgent.^137^ Hearing applications at ‘the last hour’, for instance, in these circumstances where the pregnant women are in the latest stages of pregnancy,^138^ would no doubt scupper hopes of full patient participation. Judges are left with no option but to hear these applications seeking anticipatory declarations, but they are often heard in urgent circumstances, which is done at the expense of patient empowerment by disregarding the voices of those at the heart of these cases. This again shows how these types of anticipatory declaration applications have become a new creative method to manage the temporal issues that test and push the boundaries of mental capacity law.

## V. THE TEMPORAL CHALLENGES: EMERGENCY PROVISIONS VERSUS ADVANCE PLANNING

As argued so far, the bodily autonomy of the patient is ‘entangled’ in the institutional concerns with ‘safety netting’ and potential liabilities, which are themselves complicated by the associated temporal issues of urgency that lie at the heart of these cases. A recent surge of interest in temporality has emerged in medico-legal commentary, for instance, Garland and Travis have explored how health, psychosocial care, and legal institutions present distinct temporal interpretations of the same body in terms of intersex embodiment.^139^ In a similar fashion, this section discusses how temporal factors can shape the trajectory of these cases. As will be argued, NHS Trusts are thrust into thorny territory due to the lack of legal clarity on how to approach matters where a future loss of capacity looks likely. For example, obstacles relating to temporality have caused the Trusts in these cases to receive criticism for bringing applications both too late^140^ and too cautiously.^141^ Criticism of Trusts for their handling of capacity issues relating to obstetric decisions has also occurred, in general, quite infamously in the case of *Re CA*.^142^ In his judgment, Baker J condemned the ‘failure’ of the NHS Trust for bringing an application for an order authorizing a caesarean section less than 2 weeks before the patient’s expected due date, which the judge described as having caused an ‘extremely unsatisfactory situation’.^143^ Baker J’s sentiments have been aptly reflected in the title of McCombe’s article: ‘Your lack of planning does not constitute my emergency’.^144^ Due to the questions around whether these patients in each case were considered to be in ‘emergency’ circumstances or not, this has paved the way for this new avenue of requesting anticipatory declarations from the CoP. It is clear that NHS Trusts have been faced with a choice: to either delay and see if the situation presents as an emergency, thereby invoking Sections 5 and 6 of the MCA, or to attempt to head off any potential liability claims by seeking an extension of the MCA (ie an anticipatory declaration) and bringing the jurisdiction forward in time to those who *may* lose capacity in the future. This pinpoints the core temporal conflicts that have a direct effect on how capacity law operates and transpires. Along with this, these cases raise questions around the concept of ‘emergency’ and how the boundaries of this can be stretched and shifted (by institutions) to fit in with institutional needs.

In the case sample detailed here, the judges in four out of the six obstetric cases criticized the timing of the applications. In *GSTT v R*, Hayden J stated that he ‘deprecate[d] the delay in bringing the application’^145^; in *North Middlesex University Hospital v SR*, Gollop KC reiterated the fact that where an application is warranted, ‘delay must be avoided’^146^; in *North Middlesex University Hospital v MB*, Roberts J stated she could ‘see no good reason why this application could not have been issued much sooner’^147^; and in *Shrewsbury and Telford Hospital v T*, Lieven J stated the application ‘should have been made much earlier’.^148^ Interestingly, however, in this latter case, Lieven J found that even by the time the application was heard in court, the risk of the patient losing capacity was only ‘small’, which was not enough to warrant the anticipatory declaration sought.^149^ Moreover, the judge made it clear that she believed there were alternative ways of managing the situation in question, namely: inviting the patient to make an advance statement of wishes and feelings, or, if there was a true emergency, relying on the doctrine of necessity.^150^ She warned: ‘There needs to be some caution about turning what are in truth medical decisions into legal ones’.^151^ As has been noted elsewhere, however,^152^ the European Court of Human Rights in *Glass v United Kingdom*^153^ found an Article 8 European Convention on Human Rights violation where an NHS Trust had failed to seek a court order and instead treated a patient without consent on an emergency basis.^154^ Thus, it appears as if Trusts are in an undesirable position. The temporal pressures in such situations, for instance, the amount of time in which a pregnant person is in labour, often indicate that NHS Trusts are under significant stress to just take some form of action, whatever that may be.

An issue that naturally requires consideration is whether any other legal processes can be relied upon by NHS Trusts when a loss of capacity looks likely. It becomes clear from this consideration that the timing of applications and the temporality of the general circumstances have an influence on the legal options available. For instance, as stated above, Lieven J in *Shrewsbury and Telford Hospital v T* and Mostyn J in *Somerset v Amira* argued that Trusts should be able to rely on the defence of necessity when a pregnant person loses capacity during labour, as this could be regarded as an emergency.^155^ Sections 5 and 6 of the MCA codify the common law doctrine of necessity^156^ in the care or treatment of individuals deemed to lack capacity. These sections pertain to an act of treatment or care in an emergency situation whereby, if the treating body, before performing the act, ‘takes reasonable steps to establish whether P lacks capacity in relation to the matter in question’,^157^ and when performing the act, ‘reasonably believes that P lacks capacity in relation to the matter’,^158^ and ‘that it will be in P’s best interests for the act to be done’,^159^ no liability will be incurred. Mostyn J in *Somerset v Amira* stated he admitted to being ‘at a loss’ as to why this is not ‘routinely used…to obtain immunity from a later complaint by P about an act done in connection with her care or treatment’ because ‘it is specifically legislated for in the Act’.^160^ Also in *Somerset v Amira*, Mostyn J discussed the possibility of making an interim order under Section 48 of the MCA.^161^ This power allows the court to decide what happens to an individual ‘pending the determination’ of the application, in other words, without making a final declaration on the person’s capacity, but where there is ‘reason to believe’ that person lacks capacity.^162^ Mostyn J noted that the nature of interim orders means ‘there is a real prospect… that incapacity will not be proved at the final hearing’ and that this is ‘as close as the Act gets to extending its powers to people with capacity’.^163^ As Hayden J in *DP (By His Accredited Legal Representative) v London Borough of Hillingdon*^164^ stated, ‘Section 48 is a permissive provision in the context of an *emergency* jurisdiction which can only result in an order being made where it is identifiably in P’s best interests’.^165^ However, there is doubt as to whether one can label a situation as an emergency where loss of capacity is expected. The fact that emergency provisions are proposed, by a judge (acting within their institution), as possible legal recourse for healthcare providers in these situations suggests a shifting and boundary-pushing of the concept of ‘emergency’ to adhere to another institutional need (protection from liability). It is these nuances that would go unnoticed if one were to take a surface view of anticipatory orders, but a more institutional, spatial perspective illuminates the subtleties, including temporal factors, that clearly shape and mould the way the law is steered. Nonetheless, it is difficult to agree with Mostyn J’s extension of emergency in these circumstances. For instance, in all the cases in the sample, anticipatory orders were sought *because* the Trusts felt a loss of capacity was likely during labour. A true emergency in the context of Section 5 of the MCA cannot be anticipated. An anticipated loss of capacity during labour is, instead, a foreseeable event that requires contingent planning. The question which flows from this, then, is whether contingent plans can be made without the court’s input and without seeking an anticipatory declaration.

The most common way of planning for any future losses of capacity is making an advance decision to refuse treatment (ADRT) under Section 24 of the MCA. ADRTs are legally binding but they are restricted to decisions *refusing* treatment.^166^ An advance statement of wishes and feelings could also be formulated which would give any treating team a clearer understanding of a patient’s capacitous preferences at a time when they lose capacity.^167^ However, these are not legally binding and thus do not necessarily prevent an overriding of what is written in the statement. The problem is that in all the cases from the sample, the Trusts made applications for anticipatory declarations because they were worried that the patients would lose capacity to *consent* to caesarean sections. In other words, the Trusts needed a court order in lieu of the patient’s abilities to give *advance consent*, especially if restraint or a deprivation of liberty, was likely. The evidence on whether an individual can give advance consent is unclear and ambiguous, as in its White Paper on reforming the MHA, the Government stated: ‘the principle that people should be able to make decisions which will endure in the event of future incapacity, including advance consent, is already recognised in law.’^168^ Advance consent was not enacted in the Mental Capacity (Amendment) Act 2019, however,^169^ nor is it clear what other legal authority recognizes advance consent. Interestingly, the new draft MCA Code of Practice and Liberty Protection Safeguards made proposals that advance consent to specific arrangements, which could amount to a deprivation of liberty, should be possible.^170^ As the final code has not yet been published and has in fact been delayed,^171^ it is unclear at present whether it is possible for a patient to provide consent in advance to procedures such as a caesarean section in the event that they lose capacity. Opinions on advance consent are not altogether favourable,^172^ but such a legal route could avoid the need to apply to the CoP for draconian and invasive anticipatory orders. At least, advance care plans (including advance consent) are decided upon and created by patients themselves. In this sense, they would give patients more control over what would essentially be performed upon them at a time when they lose capacity.

The point remains, however, that the evidence indicates that NHS Trusts may feel compelled to seek anticipatory orders from the CoP when a loss of capacity looks likely, due to the temporal constraints in these situations and the lack of other legal avenues available. Relying on the defence of necessity would appear problematic because of the anticipatory nature of these situations, and similarly, relying on advance care plans and advance consent is not feasible due to the uncertainty surrounding their legal status. Moreover, it has been argued in this section that these applications for anticipatory declarations force the courts to attempt to manage the delicate temporal aspects of these anticipatory cases whilst still adhering to mental capacity legislation. As such, issues of temporality can be considered to influence the way in which the law operates.

## VI. CONCLUSION

This article has drawn together a sample of cases in which an unnerving trend has emerged for the seeking of anticipatory declarations in relation to the potential future loss of capacity in pregnant people. In analysing the case sample, this article has adopted a relational and spatial approach and has viewed the facts of these cases from both a patient empowerment and an institutional perspective. From the position of the patient, the use of anticipatory declarations cannot be seen as a positive. Although the wishes and feelings of the patients in most of the cases were given weight in the court’s final declarations on best interests, so were the interests of the foetus these patients were carrying. In such a contentious area of medical law, where there is a constant balancing of patient rights versus foetal interests,^173^ there is a need for the courts and Trusts to prioritize and facilitate hearing directly from the person at the heart of proceedings, something which did not occur in any cases in the sample. This article has looked beyond the patient, however, and has considered the spatial and temporal factors attached to these contingent declarations through an institutional lens. A review of the sample under these terms revealed that NHS Trusts are ultimately affected by both the looming threat of liability claims that could come their way and the lack of consensus on the best approach in situations where a future loss of capacity appears likely to occur. Furthermore, the article discussed the temporal challenges associated with these types of issues and found that, because of the clashing emergency versus non-emergency interpretations of a loss of capacity during labour, NHS Trusts are conflicted on how to approach matters within the confines of the MCA. As such, it has been argued that focusing beyond the patient’s perspective and considering things from a broader, spatial standpoint can trigger a rethinking of the concept of mental capacity. Scholars in other fields have noted the problems with simply remaining within the borders of binary constructions, for instance, in terms of disability, the disabled/non-disabled dichotomy could lead to issues with inclusion and a denial of ‘full participation and full citizenship for disabled people’.^174^ Similarly, there is a significant amount of commentary on the dismantling of gender norms,^175^ interlinking with scholarship that highlights the boundaries imposed on race,^176^ class,^177^ and identity.^178^ Rejecting legal norms that are so embedded in institutions, relationships, and other normative structures becomes a difficult task when they remain accepted by both the formal legal processes set out by the law and by those complying with or following those legal processes.^179^ This article has acted as a cue to be mindful: that only focusing on simply the individual responses to mental capacity issues may not be the answer, but instead looking to challenge the actual formation and process of mental capacity as a construct can possibly open up new ways of reimagining responses. There is not the room in this article to fully explore what these new responses could be; nor is the answer clear-cut. Yet, it is hoped that this article sheds further light on the CoP’s continuing creative efforts to effectively manage the relational, spatial, and temporal sensitivities in cases that do not necessarily fit into the capacity/incapacity blueprint, whilst at the same time appearing to maintain full compliance with the MCA framework. As a result, the legitimacy of the MCA itself cannot fall under the radar and be left untouched; it requires much further exploration, deconstruction, and debate.

## Footnotes

1 This article uses ‘contingent declaration’ and ‘anticipatory declarations’ interchangeably throughout.

2 The terms ‘pregnant people’ and ‘pregnant person’ are used throughout this article (when discussing this group of people generally) to be inclusive of those who carry pregnancies but do not identify as women. However, when referring to parties to the proceedings in cases discussed, I will use the term ‘pregnant woman’ as each person in the cases identify as women.

3
*The Shrewsbury and Telford Hospital NHS Trust v T & Anor* [2023] EWCOP 20 at [24].

4 S Halliday, ‘Pregnancy and Severe Mental Illness: Birth Choices, Best Interests and the Untapped Potential of Advance Decisions’ (2023) 24 Medical Law International 1, 2.

5 NHS England, ‘Three Year Delivery Plan for Maternity and Neonatal Services’ (March 2023) [1.3] <https://www.england.nhs.uk/wp-content/uploads/2023/03/B1915-three-year-delivery-plan-for-maternity-and-neonatal-services-march-2023.pdf> accessed 20 February 2024.

6 There is an abundance of literature on this topic, some of which will be discussed further into this article. For a brief exploration, see K Coxon, J Sandall, and NJ Fulop, ‘To What Extent are Women Free to Choose Where to Give Birth? How Discourses of Risk, Blame and Responsibility Influence Birth Place Decisions’ (2014) 16 Health, Risk & Society 51.

7 J Harrington, *Towards a Rhetoric of Medical Law* (Routledge 2016); E Cloatre, ‘Towards a Rhetoric of Medical Law’ (2018) 12 *Law and Humanities* 93; A Ruck Keene and others, ‘Mental Capacity—Why Look for a Paradigm Shift?’ (2023) 31 Medical Law Review 340.

8 B Clough, *The Spaces of Mental Capacity Law: Moving Beyond Binaries* (Routledge 2021).

9 ibid.

10 One further case, *Re RH* [2022] 4 WLUK 287, was found to be relevant but no judgment was available, only a case digest on Westlaw. This has been excluded from my analysis.

11 [2019] EWCOP 24.

12 [2020] EWCOP 4.

13 [2021] EWCOP 58.

14 [2023] EWCOP 23.

15
*Shrewsbury* (n 3).

16 [2023] EWCOP 25.

17
*Wakefield Metropolitan District Council & Anor v DN & Anor* [2019] EWHC 2306.

18
*A Local Authority v PG & Ors* [2023] EWCOP 9.

19
*A County Council v KK & Ors (Rev 1)* [2020] EWCOP 68.

20 It is of note that not all CoP judgments are reported, thus there may be some further cases unknown and which have therefore not been included in the sample.

21
*CD* (n 11); *GSTT* (n 12); and *SR* (n 13).

22
*MB* (n 14); *Shrewsbury* (n 3); and *Somerset* (n 16).

23
*MB* (n 14) [32] and *Somerset* (n 16) [59].

24
*Shrewsbury* (n 3) [24].

25
*Re T (Adult: Refusal of Medical Treatment)* [1992] 4 All ER 645, [652].

26 ibid [653].

27
*Re MB (Medical Treatment)* [1997] 2 FLR 426.

28 Mental Capacity Act 2005, s 4.

29 ibid s 2(1).

30 ibid s 3(1); see also *A Local Authority v JB (Rev1)* [2021] UKSC 52 [76].

31 ibid s 1(2).

32 ibid s 1(3).

33 ibid s 1(4).

34
*Heart of England NHS Foundation Trust v JB* [2014] EWCOP 342 [43]: ‘JB undoubtedly has a disturbance in the functioning of her mind in the form of paranoid schizophrenia… but that it has not been established that she thereby lacks the capacity to make a decision about surgery for herself.’

35 MCA (n 28) s 2.1: ‘because of an impairment of, or disturbance in the functioning of, the mind of brain’.

36 For instance, in *Royal Borough of Greenwich v CDM* (Rev 1) [2019] EWCOP 32, where the patient was said to have fluctuating capacity, because this is an issue not mentioned in the Act, Newton J created a new ‘micro/macro’ standard to help them distinguish the ‘bigger picture’ decisions from intricate individual ones, allowing him to make a declaration that the patient lacked capacity over the ‘macro’ decision, that is her diabetes treatment. For further discussion on this, see M Curtice, ‘Fluctuating Capacity: The Concept of Micro and Macro Decisions’ (2020) 26(4) BJ Psych Advances 238.

37 [2014] EWCOP 30.

38 [2014] EWCOP 30: Practice Note.

39
*FG* (n 37) [73].

40
*GSTT* (n 12) [36] per Hayden J.

41
*CD* (n 11) [16], ibid [7].

42
*GSTT* (n 12) [29]; MCA (n 28) s 15(1)(c).

43 ibid [36].

44 S Fovargue, ‘Anticipating Issues with Capacitous Pregnant Women: United Lincolnshire NHS Hospitals Trust v CD [2019] EWCOP 24 and Guys and St Thomas’ NHS Foundation Trust (GSTT) and South London and Maudsley NHS Foundation Trust (SLAM) v R [2020] EWCOP 4’ (Commentary) (2020) 28 Medical Law Review 781, 788.

45 MCA (n 28) s 2(1); ibid 788.

46 S Fovargue (n 44) 789 (own emphasis added).

47
*C (Capacity to Access the Internet and Social Media)* [2020] EWCOP 73 [8].

48 Clough (n 8) 65.

49 ibid.

50
*CD* (n 11) [16].

51 ibid [6].

52 S Fovargue, ‘In Whose Best Interests? Childbirth Choices and other Health Decisions’ (2021) 137 Law Quarterly Review 1, 18.

53 S Villarmea, ‘When a Uterus Enters the Room, Reason Goes out the Window’ in C Pickles and J Herring (eds) *Women’s Birthing Bodies and the Law: Unauthorised Intimate Examinations, Power and Vulnerability* (Hart 2020) 69.

54 ibid.

55
*CD* (n 11); *GSTT* (n 12).

56
*SR* (n 13); *MB* (n 14); *Shrewsbury* (n 3); *Somerset* (n 16).

57 S Fovargue (n 52) 35.

58 SR Baker and others, ‘“I Felt as though I’d been in Jail”: Women’s Experiences of Maternity Care during Labour, Delivery and Immediate Postpartum’ (2005) 15 Feminism & Psychology 315, 323–332.

59 T Anderson, ‘The Misleading Myth of Choice: The Continuing Oppression of Women in Childbirth’ in M Kirkham (ed), *Informed Choice in Maternity Care* (Palgrave Macmillan 2004) 257–264.

60 R Gregg, ‘“Choice” as a Double-edged Sword: Information, Guild and Mother-blaming in a High-tech Age’ (1993) 20 Women Health 55.

61 JA Lothian, ‘Choice, Autonomy and Childbirth Education’ (2008) 17 The Journal of Perinatal Education 35; ML Crossley, ‘Childbirth, Complications and the Illusion of “Choice”: A Case Study’ (2007) 17 Feminism & Psychology 543.

62 SR Baker and others (n 58) 335.

63 ibid.

64 See M Brazier and J Miola, ‘Bye-Bye Bolam: A Medical Litigation Revolution’ (2000) 8 Medical Law Review 85; E Jackson, ‘From “Doctor Knows Best” to Dignity: Placing Adults Who Lack Capacity at the Centre of Decisions About Their Medical Treatment’ (2018) 81 Modern Law Review 247.

65 S Halliday, *Childbirth, Vulnerability and Law: Exploring Issues of Violence and Control* (Routledge 2019) 183.

66 ibid.

67 A capacitous pregnant woman can ‘for religious reasons, other reasons, for rational or irrational reasons or for no reason at all, choose not to have medical intervention, even though… the consequence may be the death or serious handicap of the child she bears, or her own death’ per Butler-Sloss LJ in *Re MB* (n 27) [436]–[437].

68 MCA (n 28) s 4.

69 M Fox and J Lindsey, ‘Health Law, Medicine and Ethics: Are Women More Likely to Have Their Healthcare Decisions Overruled?’ in R Auchmuty (ed), *Great Debates in Gender and Law* (Bloomsbury Publishing Plc 2018) 123–124.

70 EC Romanis, ‘Why the Elective Caesarean Lottery is Ethically Impermissible’ (2019) 27 Health Care Analysis 249, 255; Z Mahmoud and EC Romanis, ‘On Gestation and Motherhood’ (2022) 31 Medical Law Review 109.

71 Romanis ibid 257.

72 R Brione, ‘To What Extent does or should a Woman’s Autonomy Overrule the Interests of her Baby? A Study of Autonomy Related Issues in the Context of a Caesarean Section’ (2015) 21 The New Bioethics 71, 83.

73
*CD* (n 11) [4].

74 ibid.

75 ibid [8].

76
*GSTT* (n 12) [4].

77 ibid [42] and [63].

78
*MB* (n 14) [33].

79 Mahmoud and Romanis (n 70) 132.

80 S Halliday, *Autonomy and Pregnancy: A Comparative Analysis of Compelled Obstetric Intervention* (Routledge 2016) 2.

81
*CD* (n 11) [7].

82 ibid [16].

83
*SR* (n13) [9].

84 ibid [48]–[50].

85
*MB* (n 14) [30].

86 ibid [33].

87
*Somerset* (n 16) [5].

88 ibid [68].

89
*GSTT* (n 12) [58]: ‘it was not possible for the Official Solicitor to speak with R to achieve greater clarity as to her wishes and feelings’.

90 ibid [56].

91 ibid [66]–[67].

92 ibid [62].

93 ibid [63].

94 Mahmoud and Romanis (n 70) 131.

95
*Shrewsbury* (n 3) [25].

96 ibid [23].

97 ibid.

98
*Rotherham and Doncaster and South Humber NHS Foundation Trust v NR & Anor* [2024] EWCOP 17 [52].

99 39 Essex Chambers, ‘Rotherham and Doncaster and South Humber NHS Foundation Trust v NR & Anor’ (8 March 2024) <https://www.39essex.com/information-hub/case/shrewsbury-and-telford-hospital-nhs-trust-v-t-and-midlands-partnership-nhs> accessed 6 July 2024.

100
*Shrewsbury* (n 3) [23].

101 C Mackenzie and N Stoljar (eds), *Relational Autonomy* (OUP 2000).

102 See also, C Mackenzie, ‘Relational Autonomy, Normative Autonomy and Perfectionism’ (2008) 39 J Soc Phil 512; C Kong, *Mental Capacity in Relationship* (CUP 2017).

103 The MCA was once labelled as the ‘gatekeeper for autonomy’ with access to autonomy controlled by a diagnosis of capacity [see M Donnelly, *Healthcare Decision-Making and the Law: Autonomy, Capacity and the Limits of Liberalism* (CUP 2010) 90]; however, this has been contested by the argument that some capacitous choices are not genuinely autonomous as making a ‘dangerous’ but capacitous one may leave a person without protection from vulnerability [see J Herring, *Vulnerable Adults and the Law* (OUP 2016) 64].

104 I Kant, ‘Groundwork of the Metaphysics of Morals’, in M Gregor (ed) *Kant: Groundwork of the Metaphysics of Morals* (CUP 2012) 1–76.

105 Mackenzie and Stoljar (n 101) 4.

106 Clough (n 8) 11–27.

107 National Maternity Review, ‘Better Births: Improving outcomes of maternity services in England: A Five Year Forward View for Maternity Care’ (22 February 2016) [3.32] <https://www.england.nhs.uk/wp-content/uploads/2016/02/national-maternity-review-report.pdf> accessed 20 February 2024; HC, Health and Social Care Committee, ‘NHS Litigation Reform’: Thirteenth Report of Session 2021-22 (April 2022) [72] <https://committees.parliament.uk/publications/22039/documents/163739/default/> accessed 21 February 2024.

108 HC, Health and Social Care Committee, ‘The Safety of Maternity Services in England’: Fourth Report of Session 2021–2022 (July 2021) [90] <https://committees.parliament.uk/publications/6578/documents/73151/default/> accessed 28 February 2024.

109 S Elaraby and others, ‘Behavioural Factors Associated with Fear of Litigation as a Driver for the Increase Use of Caesarean Sections: A Scoping Review’ (2023) 13 British Medical Journal 1; J Robertson and A Thomson, ‘A Phenomenological Study of the Effects of Clinical Negligence Litigation on Midwives in England: The Personal Perspective’ (2014) 30 Midwifery 121.

110 E Cave, ‘Protecting Patients from their Bad Decisions: Rebalancing Rights, Relationships and Risk’ (2017) 25 Medical Law Review 527, 553.

111
*CD* (n 11) [16]; *SR* (n 13) [47].

112
*GSTT* (n 12) [13].

113 ibid [17].

114
*Shrewsbury* (n 3) [23].

115
*Somerset* (n 16) [28] and [41]: ‘I admit to being at a loss as to why the ss 4B, 5 and 6 route… is not routinely used. It is specifically legislated for in the Act.’

116 ibid [41].

117 For further on this point, see C Kong and others, ‘The “Human Element” in the Social Space of the Courtroom: Framing and Shaping the Deliberative Process in Mental Capacity Law’ (2022) 42 Legal Studies 715, 716.

118 R Reed-Berendt, ‘“The Baby has to Come Out”: Court-authorised Caesarean when Subject-matter and Litigation Capacity are Disputed’ (*Open Justice Court of Protection Project*, 3 March 2023) <https://openjusticecourtofprotection.org/2023/03/03/the-baby-has-to-come-out-court-authorised-caesarean-when-subject-matter-and-litigation-capacity-are-disputed/> accessed 10 January 2024.

119 ibid. The difference here, however, is that this application was not for an anticipatory declaration, it was instead for an order that the patient lacked capacity to consent to a caesarean section. It could be argued, therefore, that preference against use of an anticipatory declaration was understandable given the applicants were not seeking that outcome.

120 R Wheeler, ‘Anticipatory Declarations’ (2023) 105 The Bulletin of the Royal College of Surgeons of England 322.

121 Gov.uk, Court of Protection <https://www.gov.uk/courts-tribunals/court-of-protection> accessed 29 April 2024.

122 M Beckford, ‘Secret Court of Protection Can Order Abortions and Sterilisations of Mentally Ill Patients’ (*The Telegraph*, 28 May 2010) <https://www.telegraph.co.uk/news/health/news/7772172/Secret-Court-of-Protection-can-order-abortions-and-sterilisations-of-mentally-ill-patients.html> accessed 29 April 2024.

123 L Series and others, ‘Transparency in the Court of Protection: Report on a Roundtable’ (April 2015) <http://sites.cardiff.ac.uk/wccop/files/2015/04/Transparency-in-the-Court-of-Protection-Report.pdf> accessed 28 April 2024.

124 P′ refers to the person who is the subject of the proceedings under the CoP Rules 2017, SI 2017 No 1035, r 2.1. This article uses the abbreviation of ‘P’ in this section to reflect the literature discussed here but, where possible, will refer to the person party to proceedings as the ‘patient’ or the ‘person’.

125 L Series, P Fennell, and J Doughty, ‘The Participation of P in Welfare Cases in the Court of Protection’ (February 2017) <https://orca.cardiff.ac.uk/id/eprint/98448/1/Series%20Fennell%20Doughty%20Feb%202017%20Participation%20of%20P.pdf> accessed 29 April 2024; C Kong and others, ‘Judging Values and Participation in Mental Capacity Law’ (2019) 8 Laws 3.

126 ibid.

127 J Lindsey, *Reimagining the Court of Protection: Access to Justice in Mental Capacity Law* (CUP 2022) 189–190.

128 ibid 189.

129 ibid 192–193.

130
*SR* (n 13) [5].

131 ibid [6].

132 BM McKimmie, JM Hays, and D Tait, ‘Just Spaces: Does Courtroom Design affect how the Defendant is Perceived?’ (2016) 23 Psychiatry, Psychology and Law 885 [as cited in J Lindsey (n 127) 193].

133 J Lindsey, ‘Testimonial Injustice and Vulnerability: A Qualitative Analysis of Participation in the Court of Protection’ (2019) 28 Social & Legal Studies 450, 452–454; J Lindsey (n 127) 200.

134
*GSTT* (n 12) [58].

135 ibid [3].

136 E Flynn, *Disabled Justice? Access to Justice and the UN Convention on the Rights of Persons with Disabilities* (1st edn, Routledge 2017) 15. For more on disability, see J Craigie and others, ‘Legal Capacity, Mental Capacity and Supported Decision-Making: Report from a Panel Event’ (2019) 62 International Journal of Law and Psychiatry 160

137
*CD* (n 11); *GSTT* (n 12); *SR* (n 13); *Shrewsbury* (n 3); *Somerset* (n 16).

138 In *CD* (n 11), CD was 35 weeks pregnant; in *GSTT* (n 12), R was 39 weeks and 6 days pregnant; in *SR* (n 13), SR was 36 weeks pregnant; in *MB* (n 14), MB was 37 weeks pregnant; in *Shrewsbury* (n 3), T was 39 weeks pregnant; and in *Somerset* (n 16), Amira was due to give birth on the day of the hearing.

139 F Garland and M Travis, ‘Temporal Bodies: Emergencies, Emergence, and Intersex Embodiment’ in C Dietz, M Travis, and M Thomson (eds), *Jurisprudence of the Body* (Palgrave Macmillan 2020).

140
*Shrewsbury* (n 3) [22].

141 ibid [25].

142 [2016] EWCOP 51.

143 ibid [4].

144 K McCombe, ‘“Your Lack of Planning does not Constitute my Emergency”—Caring for Obstetric Patients with Mental Illness’ (2018) 73 Anaesthesia 807.

145
*GSTT* (n 12) [6].

146
*SR* (n 13) [28].

147
*MB* (n 14) [3].

148
*Shrewsbury* (n 3) [22].

149 ibid [24].

150 ibid [25].

151 ibid.

152 39 Essex Chambers, ‘The Shrewsbury and Telford Hospital NHS Trust v T and Midlands Partnership NHS Foundation Trust’ (23 May 2023) <https://www.39essex.com/information-hub/case/shrewsbury-and-telford-hospital-nhs-trust-v-t-and-midlands-partnership-nhs> accessed 3 March 2024.

153 [2004] ECHR 103.

154 ibid [83].

155
*Shrewsbury* (n 3) [25]; *Somerset* (n 16) [28] and [41].

156 See *Re F Mental Patient: Sterilisation)* [1990] 2 AC 1.

157 MCA (n 28) s 5(1)(a).

158 ibid s 5(1)(b)(i).

159 ibid s 5(1)(b)(ii).

160
*Somerset* (n 16) [41].

161 ibid [32].

162 MCA (n 28) s 48(a).

163
*Somerset* (n 16) [32]. Mostyn J refers to his previous judgment in *A Local Authority v LD & Anor* [2023] EWHC 1258 (Fam) in explaining this.

164 [2020] EWCOP 45.

165 ibid [62] (own emphasis added).

166 MCA (n 28) s 24.

167 NICE Guidelines, *Decision-Making and Mental Capacity* (NG108, 3 October 2018) [1.3] <https://www.nice.org.uk/guidance/ng108/resources/decisionmaking-and-mental-capacity-pdf-66141544670917> accessed 18 December 2023.

168 ‘Reforming the Mental Health Act’ (January 2021) 64 <https://assets.publishing.service.gov.uk/government/uploads/system/uploads/attachment_data/file/951398/mental-health-act-white-paper-web-accessible.pdf> accessed 18 December 2023.

169 See ‘Government’s Final Response to the Law Commission’ (14 March 2018) [43] <https://questions-statements.parliament.uk/written-statements/detail/2018-03-14/HCWS542> accessed 18 December 2023.

170 HM Government, *Mental Capacity Act 2005 Code of Practice Including the Liberty Protection Safeguards* 2022 [12.55]–[12.68] <https://assets.publishing.service.gov.uk/government/uploads/system/uploads/attachment_data/file/1061499/draft-code-of-practice.pdf> accessed 18 December 2023.

171 ‘Next Steps to put People at the Heart of Care: A plan for adult social care system reform 2023 to 2024 and 2024 to 2025’ (4 April 2023) 4 <https://assets.publishing.service.gov.uk/media/642b0270ddf8ad000cac0cb5/next-steps-to-put-people-at-the-heart-of-care_a-plan-for-adult-social-care-system-reform-2023-to-2024-and-2024-to-2025.pdf> accessed 18 December 2023.

172 For instance, see Written evidence of Roger Laidlaw provided to the Joint Committee on Human Rights (DOL0045) [12] <http://data.parliament.uk/writtenevidence/committeeevidence.svc/evidencedocument/human-rights-committee/the-right-to-freedom-and-safety-reform-of-the-deprivation-of-liberty-safeguards/written/79432.html> accessed 20 April 2024; and Written evidence of Professor Rob Heywood provided to the Joint Committee on Human Rights (DOL0043) [1] <http://data.parliament.uk/writtenevidence/committeeevidence.svc/evidencedocument/human-rights-committee/the-right-to-freedom-and-safety-reform-of-the-deprivation-of-liberty-safeguards/written/79423.html> accessed 20 April 2024, referred to in Joint Committee on Human Rights, *The Right to Freedom and Safety: Reform of the Deprivation of Liberty Safeguards* (Seventh Report of 2017-19, HC 890, HL 161) [54].

173 M Neal, ‘“Not Nothing”? The Late Term Foetus in the Court of Protection’ (*Open Justice Court of Protection Project*, 3 June 2021) <https://openjusticecourtofprotection.org/2021/06/03/not-nothing-the-late-term-foetus-in-the-court-of-protection/> accessed 22 April 2024.

174 KQ Hall, ‘New Conversations in Feminist Disability Studies: Feminism, Philosophy, and Borders’ (2015) 30 Hypatia 1, 7.

175 J Butler, *Undoing Gender* (Routledge 2004); J Lorber, *Paradoxes of Gender* (YUP 1994); D Spade, ‘Resisting Medicine, Re/modelling Gender’ (2003) 18 Berkeley Women’s Journal 15.

176 R Thomson Ford, ‘The Boundaries of Race: Political Geography in Legal Analysis’ (1994) 107 Harvard Law Review 1841.

177 U Beck, ‘Beyond Class and Nation: Reframing Social Inequalities in a Globalizing World’ (2007) 58 The British Journal of Sociology 679.

178 A Paasi, ‘Boundaries as Social Processes: Territoriality in the World of Flows’ (1998) 3 Geopolitics 69.

179 For more on this point, see N Deitelhoff and L Zimmermann, ‘Norms under Challenge: Unpacking the Dynamics of Norm Robustness’ (2019) 4 Journal of Global Security Studies 2.

